# Biochemical and phylogenetic analyses of phosphatidylinositol production in *Angomonas deanei*, an endosymbiont-harboring trypanosomatid

**DOI:** 10.1186/s13071-015-0854-x

**Published:** 2015-04-24

**Authors:** Allan C de Azevedo-Martins, João MP Alves, Fernando Garcia de Mello, Ana Tereza R Vasconcelos, Wanderley de Souza, Marcelo Einicker-Lamas, Maria Cristina M Motta

**Affiliations:** Laboratório de Ultraestrutura Celular Hertha Meyer, Instituto de Biofísica Carlos Chagas Filho, Centro de Ciências da Saúde, Universidade Federal do Rio de Janeiro, UFRJ, Avenida Carlos Chagas Filho, 343, Bloco G, Subsolo, Cidade Universitária, Ilha do Fundão, Rio de Janeiro, CEP 21941-590 Brazil; Instituto Nacional de Ciência e Tecnologia em Biologia Estrutural e Bioimagens, Rio de Janeiro, Brazil; Laboratório Nacional de Computação Científica, Av. Getúlio Vargas, 333, Quitandinha, Petrópolis, RJ CEP: 25651-075 Brazil; Department of Parasitology, Institute of Biomedical Sciences, University of São Paulo, São Paulo, Brazil; Laboratório de Neuroquímica, Instituto de Biofísica Carlos Chagas Filho, Centro de Ciências da Saúde, Universidade Federal do Rio de Janeiro, UFRJ, Avenida Carlos Chagas Filho, 343, Bloco C, Cidade Universitária, Ilha do Fundão, Rio de Janeiro, CEP 21941-590 Brazil; Instituto Nacional de Metrologia, Qualidade e Tecnologia - Inmetro, Rio de Janeiro, RJ Brasil; Laboratório de Biomembranas, Instituto de Biofísica Carlos Chagas Filho, Centro de Ciências da Saúde, Universidade Federal do Rio de Janeiro, UFRJ, Avenida Carlos Chagas Filho, 343, Bloco G, Cidade Universitária, Ilha do Fundão, Rio de Janeiro, CEP 21941-590 Brazil

**Keywords:** Phosphatidylinositol metabolism, Symbiosis, Phylogenetic analysis

## Abstract

**Background:**

The endosymbiosis in trypanosomatids is characterized by co-evolution between one bacterium and its host protozoan in a mutualistic relationship, thus constituting an excellent model to study organelle origin in the eukaryotic cell. In this association, an intense metabolic exchange is observed between both partners: the host provides energetic molecules and a stable environment to a reduced wall symbiont, while the bacterium is able to interfere in host metabolism by enhancing phospholipid production and completing essential biosynthesis pathways, such as amino acids and hemin production. The bacterium envelope presents a reduced cell wall which is mainly composed of cardiolipin and phosphatidylcholine, being the latter only common in intracellular prokaryotes. Phosphatidylinositol (PI) is also present in the symbiont and host cell membranes. This phospholipid is usually related to cellular signaling and to anchor surface molecules, which represents important events for cellular interactions.

**Methods:**

In order to investigate the production of PI and its derivatives in symbiont bearing trypanosomatids, aposymbiotic and wild type strains of *Angomonas deanei*, as well as isolated symbionts, were incubated with [^3^H]*myo*-inositol and the incorporation of this tracer was analyzed into inositol-containing molecules, mainly phosphoinositides and lipoproteins. Gene searches and their phylogenies were also performed in order to investigate the PI synthesis in symbiontbearing trypanosomatids.

**Results:**

Our results showed that the bacterium did not incorporate the tracer and that both strains produced similar quantities of PI and its derivatives, indicating that the symbiont does not influence the production of these metabolites. Gene searches related to PI synthesis revealed that the trypanosomatid genome contains an inositol transporter, PI synthase and the *myo*-inositol synthase. Thus, the host is able to produce PI either from exogenous *myo*-inositol (inositol transporter) or from *myo*-inositol synthesized *de novo*. Phylogenetic analysis using other organisms as references indicated that, in trypanosomatids, the genes involved in PI synthesis have a monophyletic origin. In accordance with experimental data, sequences for *myo*-inositol transport or for *myo*-inositol and PI biosynthesis were not found in the symbiont.

**Conclusions:**

Altogether, our results indicate that the bacterium depends on the host to obtain PI.

**Electronic supplementary material:**

The online version of this article (doi:10.1186/s13071-015-0854-x) contains supplementary material, which is available to authorized users.

## Background

The Trypanosomatidae family is known worldwide, since it contains pathogenic species from genera *Trypanosoma* and *Leishmania*, which affect human health and the economy [1]. However, most protozoa in this family are non-pathogenic to humans and inhabit only invertebrate hosts during the whole life cycle. Between these monoxenics, six species, including *Angomonas deanei*, maintain an obligatory symbiotic relationship with an intracellular bacterium [2,3]. The mutualistic co-evolution between these primitive partners makes this relationship an excellent model to study the origin of organelles in the eukaryotic cell [4]. Symbiosis in trypanosomatid protozoa has a monophyletic origin and the bacterium is phylogenetically related to the Alcaligenaceae family of the Betaproteobacteria class, thus belonging to the Gram-negative group [2,3,5]. Treatment of trypanosomatids with chloramphenicol generated an aposymbiotic strain, which can be used as a useful tool to understand how the symbiont influences the host [4]. Previous studies revealed an intense metabolic exchange between the symbiotic bacterium and the protozoan, which is reflected in an enhanced growth rate and low nutritional requirements of symbiont-bearing trypanosomatids when compared to the aposymbiotic ones [6-9]. Actually, it has been demonstrated that the symbiotic bacterium completes several metabolic routes of the host cell, such as those involved in the production of amino acids, vitamins, and heme [10-13]. Furthermore, the presence of the symbiont is related to alterations in trypanosomatid ultrastructure [14], surface charge [15], and carbohydrate plasma membrane composition [16].

The symbiont presents an envelope composed of two membrane units and a reduced cell wall, which does not allow the symbiont either to survive or to replicate when isolated from the host [17]. Although phosphatidylcholine (PC) is a major component of symbiont membranes, once isolated the bacterium produces very low amounts of this phospholipid. Considering the host’s phospholipid composition, *A. deanei* has PC as a major constituent, followed by phosphatidylethanolamine (PE) and PI. Interestingly, comparisons between wild type and the aposymbiotic strains indicate that the presence of the symbiont is correlated with an increase in phospholipid production [18]. Accordingly, it has been reported that intracellular bacteria may complement some steps of the PI metabolism from the host, as already observed in plants as well as in animal cells infected by pathogenic prokaryotes [19,20].

In trypanosomatids, phospholipids are not obtained directly from the environment (host or medium), but synthesized using the common headgroups (such as choline, ethanolamine, inositol) and diacylglycerol. In *T. brucei*, the genes responsible for *de novo* biosynthesis of all phospholipids were identified [21,22]. The biosynthesis of PI in trypanosomatids occurs by condensation of the headgroup, in this case *myo*-inositol, with cytidine diphosphate-diacylglycerol (CDP-DAG). In the trypanosomatid *Leishmania donovani*, the uptake of *myo*-inositol occurs through an inositol/H^+^ symporter, which belongs to the glucose transporter superfamily [23,24], while in *T. cruzi myo*-inositol transport occurs through an inositol/Na^+^ symporter [25]. Similar to mammals, in trypanosomatids PI synthase (PIS) catalyzes the production of PI from CDP-DAG and *myo*-inositol, with the reaction taking place in the endoplasmic reticulum (ER) and in the Golgi apparatus [21,22]. While all eukaryotes are able to synthesize PI, most prokaryotes do not present this ability. Few prokaryotes present a PIS, which was probably obtained by lateral gene transfer from Archaea [26]. In these organisms, the production of PI occurs as follows: first, phosphatidylinositol monophosphate (PIP) is produced from inositol phosphate and cytidine monophosphate-phosphatidyl; then, PIP releases an inorganic phosphate (Pi), thus generating PI [27].

PI has an important role in cell signaling in trypanosomatids, as well as in other eukaryotic cells, being the precursor of important molecules such as phosphorylated inositol lipids, also called phosphoinositides [28,29]. Mainly located in the cytosolic layer of membranes, PI can be sequentially phosphorylated by different specific kinases (PI-K). When PI-4,5-bisphosphate is formed, it can be hydrolyzed by the PI-phospholipase C (PI-PLC) yielding inositol triphosphate, which is released into the cytoplasm, and DAG [30]. Some PI-Ks were already identified in the genome of trypanosomatids and also by biochemical activity [21,22].

Phosphatidylinositol and phosphoinositides also participate in the establishment and maintenance of symbiotic relationships, as observed in symbiosis between fungi and plants [31] and between bacteria and plants [19,32]. It was previously demonstrated that the symbiotic bacteria *Rhizobium leguminosarum* presents a phosphotransferase whose activity increases the PI 4-phosphate content of the plant host, which is essential to the nodulation process [19]. In the symbiosis between *Medicago truncatula* and *Rhizobium* bacteria, nodulation is stimulated by the production of bacterial Nod factors, thus promoting the activation of calcium spikes in the host in a phosphoinositide-dependent signaling [32]. Furthermore, it has also been reported that intracellular prokaryotes, such as pathogenic bacteria, can promote alterations in the PI, PIP, and PIP_2_ content in the host [20].

In this work, we investigated the production of PI, PIP, and phosphatidylinositol biphosphate (PIP_2_) in *A. deanei* and its aposymbiotic strain in order to verify whether the bacterium influences the PI metabolism of the protozoan host. Furthermore, the enzymes involved in PI biosynthesis were also investigated, at the genome level and phylogenetically, to provide further information about this metabolic pathway in the trypanosomatid family.

## Methods

### Cell growth and the obtainment of endosymbiont fraction

Wild type (Wt) and aposymbiotic strains of *A. deanei* were grown at 28°C in Warren's culture medium [33] supplemented with 10% fetal calf serum. In all assays, both strains were cultivated for 24 hours, which corresponds to the exponential growth phase. The endosymbiont fractions were obtained as described in [18].

### [^3^H]*myo*-inositol incorporation and labeling of the PI

*A. deanei* PI was metabolically labeled as follows: log phase cells were incubated with 1 μCi/mL [^3^H]-*myo*-inositol in culture medium for 24 h. Protozoa were then washed 3 times with PBS, with centrifugation (1,800 × g), and the cell pellet was quantified by protein concentration and used for lipid extraction as described below. The isolated symbiont fraction was also incubated with the radiolabeled inositol. The fraction was resuspended in cell growth medium containing 1 μCi/mL [^3^H]-*myo*-inositol for 1 or 3 h at 28°C. After incubation, the fraction was washed 3 times with PBS, with centrifugation (1,800 × g), and then homogenized. Protein concentration was quantified for the different assays.

### Lipid extraction, separation, and identification of phospholipids

The methodology previously described by our group [18] was adopted for lipid extraction and separation. After identification of the phospholipid by thin layer chromatography (TLC), plates were dried in a fume hood and the lipid spots were visualized by exposure to iodine vapors. Autoradiography of the TLC plates was carried out using X-ray film (Kodak T-Mat) at −70°C for 10 days, and the film was developed following the manufacturer’s specifications. For the identification of radiolabeled PI spots, two methods were adopted: in the first, plates were exposed to a ^3^H-sensitive screen in a cassette stored at −20°C for 10 days and the radioactivity was then detected in Storm Phosphor Image. In the second method, PI spots identified by exposure to iodine vapor were scraped off the plate and put into vials containing scintillation solution. The radioactivity was measured using a Beckman counter.

### Chemicals

The solvents, plates and other reagents used in this work for extraction, separation, and identification of phospholipids by thin layer chromatography were obtained from Merck. [^3^H]-*myo*-inositol was purchased from Perkin Elmer. All reagents were high purity grade. Deionized water was used to prepare all media and solutions.

### Statistical analysis

The PI quantifications are expressed as mean ± standard error (±SE), using one-way ANOVA followed by post-hoc Tukey's test for multiple comparisons. Data were considered statistically significant when *p* < 0.05. Although experiments considered a starting concentration of 10^7^ cells/mL, PI measures were normalized by protein quantification.

### Genomic analysis

For genomic analysis, data from *A. deanei* and its corresponding symbiotic bacterium were obtained from our previous publication where DNA extraction and sequencing, followed by gene calling and the functional annotation, were described [5]. The assembly and associated annotations are available in NCBI's GenBank database under accession number ATMG00000000. In this work, sequences of interest are identified according to their protein identifiers. Reference sequences used in the present genomic analyses were selected from phylogenetically related organisms, as follows: *Leishmania major* and *Leishmania donovani* for comparisons with *A. deanei*, and *Mycobacterium tuberculosis* RGTB327 and *Methanothermobacter thermautotrophicus* (str. Delta H, all available from GenBank) for comparisons with the symbiotic bacterium.

### Phylogenetic analysis

To ensure wide representation of taxa from all branches of life, candidate sequences for phylogenetic analyses were selected by similarity searches of the *A. deanei* protein sequence against the whole NCBI non-redundant (nr) database (maximum E-value cutoff of 1E-10), as previously described [3]. New trypanosomatid sequences used in phylogenetic analysis were deposited in GenBank (KP689381- KP689400). The resulting datasets were aligned by Muscle v. 3.8.31 [34], ambiguously aligned positions were removed by Gblocks [35] using the “with half” option for gap treatment, and maximum likelihood phylogenetic analyses were performed by RAxML v. 8.0.24 [36], using the WAG substitution model [37], gamma-distributed rate heterogeneity categories, and empirical residue frequencies. Trees were edited and drawn in TreeGraph2 [38] and Dendroscope [39], with cosmetic adjustments done in Inkscape (http://inkscape.org).

## Results

### Phosphoinositide formation in *A. deanei*

**Figure 1:**
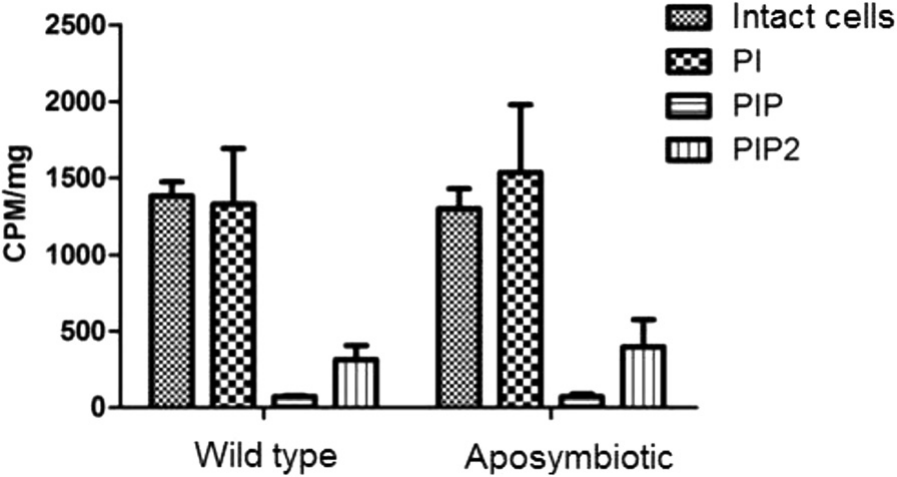
*A. deanei* synthesis of PI and its derivatives after incorporation of [^3^H]*myo*-inositol. Wild type and aposymbiotic cells presented similar quantities of PI, PIP and PIP2. The graph represents the mean CPM/mg of protein ± SE of five independent experiments after 24 hours of incubation with radiolabeled inositol.

Wild type and aposymbiotic strains of *A. deanei* were grown in culture medium containing [^3^H]*myo*-inositol in order to quantify the incorporation of this tracer in PI and in phosphoinositides. There is no significant differences in the incorporation of exogenous [^3^H]-*myo*-inositol into PI, PIP and PIP_2_ (*p* > 0,05 in all tests) after comparing wild type and aposymbiotic strain of *A. deanei* (Figure 1). The wild type cells presented a mean of 1382 ± 105 CPM/mg of protein, while the aposymbiotic cells presented 1299 ± 124 CPM/mg. The tracer was mainly incorporated in PI: 1328 ± 302 CPM/mg in wild type and 1536 ± 450 CPM/mg in aposymbiotic strain. PIP and PIP_2_ labeling were very low when compared with PI: wild type cells presented 70 ± 7 CPM/mg for PIP and 314 ± 91 CPM/mg for PIP_2_, while aposymbiotic cells presented similar values for PIP (70 ± 9 CPM/mg) and for PIP_2_ (397 ± 193 CPM/mg) (Figure 1).

**Figure 2:**
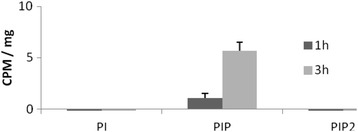
Isolated symbionts were incubated with [^3^H]*myo*-inositol in order to verify the synthesis of PI, PIP, and PIP2. The graph represents the mean CPM/mg of protein ± SE of five independent experiments using symbionts incubated for 1 h or 3 h with the radiolabeled tracer. No significant radioactivity values were observed (the highest radioactivity inositide, PIP, presented less than 6 CPM/mg).

To analyze the [^3^H]*myo*-inositol incorporation in the symbiont, the *A. deanei* wt strain was fractionated for obtainment of isolated bacteria which were incubated for one or three hours in the presence of the tracer. After extraction, identification of lipids, and radioactivity measurement, PI, PIP, and PIP_2_ spots were not labeled in the symbiont fraction (Figure 2). These results indicate that, once isolated from the host, the bacterium is not able to synthesize PI and phosphorylated PI species using exogenous *myo*-inositol as substrate.

### PI synthesis pathway

Searches for gene sequences involved in PI biosynthesis were performed in genomes of symbiont-harboring trypanosomatids and their symbionts, as well as in genomes of trypanosomatids that do not contain the bacterium. Results revealed that the host protozoan contains PIS genes (Table 1 ), indicating that PI may be synthesized from CDP-diacylglycerol and *myo*-inositol, as already reported in other trypanosomatids. Three sequences from the *A. deanei* genome deposited in GenBank (EPY39094.1, EPY39654.1 and EPY28298.1) were identified as PIS (E. C. 2.7.8.11). On the other hand, searching for PIS in the symbiont genomes using related sequences in Archaea and some bacteria (AIS) did not show any candidate genes (Table 1).PI synthesis requires exogenous *myo*-inositol or *myo*-inositol synthesized by a *de novo* pathway. The *de novo* synthesis of *myo*-inositol occurs by action of an isomerase, called L-*myo*-inositol 1-phosphate synthase (MIPS) (E. C. 5.5.1.4), that uses glucose as substrate to produce *myo*-inositol. Investigations in genomes of *A. deanei* and its symbiont revealed the presence of MIPS in the host trypanosomatid, but not in the bacterium.

**Table 1:** **Gene sequences for PI metabolism in** ***A. deanei*** **and its symbiont**

**Protein**	**Reference (organism)**	**A. deanei**	**Symbiont**
Inositol transporter	PRF 1583317 (*L. donovani*)	EPY28738.1, EPY351511, EPY29848.1	NF
PIS (eukaryotic)	XP_001684255.1 (*L. major*)	EPY39094.1, EPY39654.1, EPY28298.1	NF
PIS (prokaryotic)	AFE17439.1 (*M. tuberculosis*)	NF	NF
AIPS	O27726.1 (*M. thermatotrophicus*)	NF	NF

NF : gene not found.

Moreover, a membrane inositol transporter that would allow the entrance of exogenous *myo*-inositol into the cytoplasm was identified in the *A. deanei* genome. Represented by 3 sequences in GenBank (GenBank ID: EPY28738.1, EPY351511, and EPY29848.1), such genes have high similarity to those described in *L. donovani* (PRF 1583317). The symbiotic bacterium lacks sequences that codify for this protein. It is worth mentioning that mRNAs for PIS and inositol membrane transporter were identified in the transcriptome of *A. deanei* (submitted to publication).

**Figure 3:**
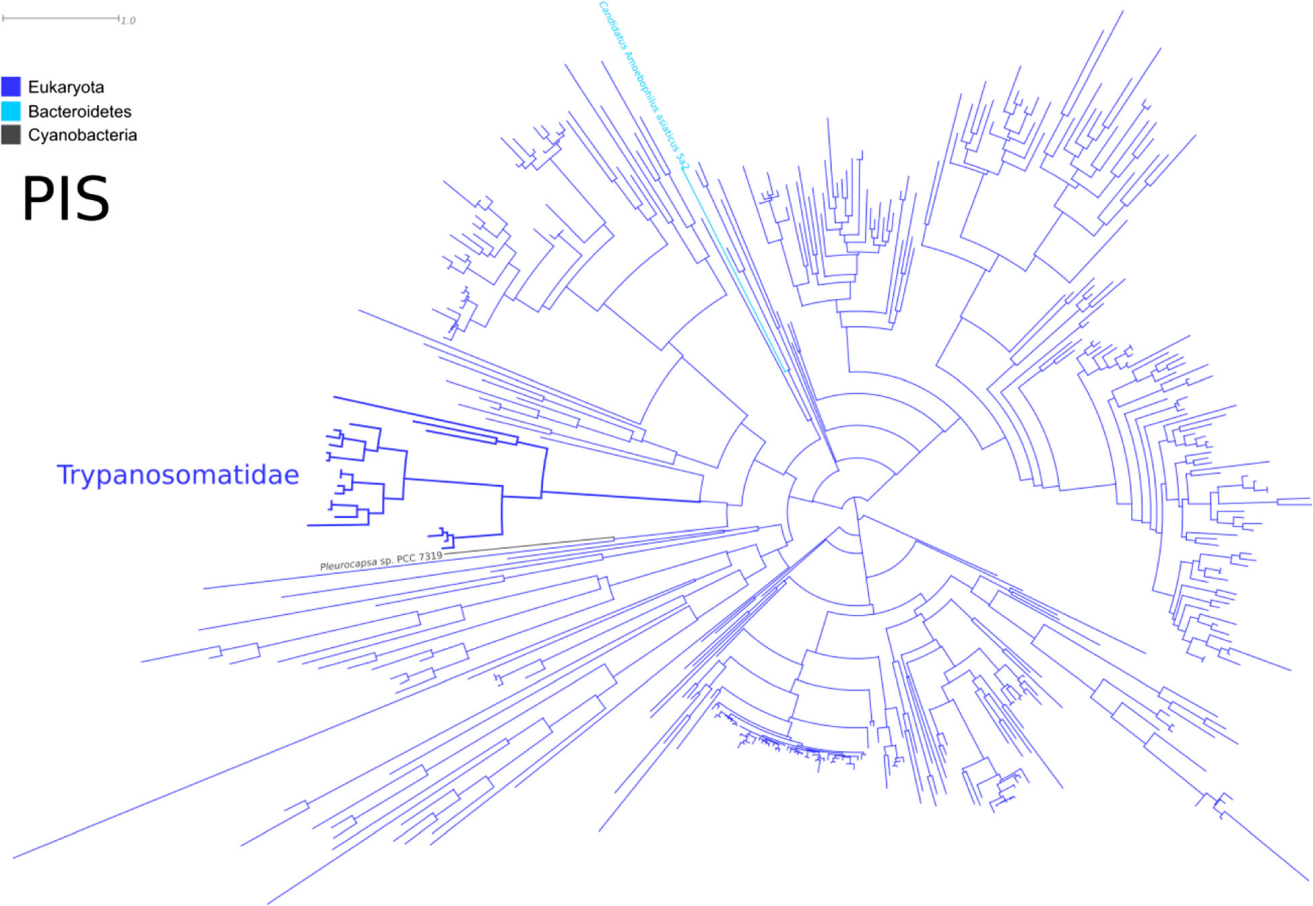
Molecular phylogenetic analysis of PIS by the maximum likelihood method. Branches are colored based on taxonomic affiliation, according to the legend on the left. The clade comprising the Trypanosomatidae family is represented by thicker branches and labeled accordingly. The only two prokaryotic sequences present in this tree are indicated by name. See Additional file 1: Figure S1 for a full version of this tree showing all names and including bootstrap support values.

Our phylogenetic analyses showed that *A. deanei* genes involved in PI production cluster with those described in other species of this genus, such as *A. desouzai*. The *Angomonas* group is inserted in the Trypanosomatidae family cluster, which also contains the regular trypanosomatids, and is placed among eukaryotes (Figure 3 and Additional file 1: Figure S1, Additional file 2: Figure S2 and Additional file 3: Figure S3). These results suggest that PI synthesis genes present a monophyletic origin in this family.

## Discussion

Our previous studies revealed that the symbiotic bacterium enhances the host phospholipid biosynthesis, including PI [19]. Here, we have shown that the bacterium genome does not contain genes for PI biosynthesis, including PI synthase, nor for inositol transporters, which were previously described in few prokaryotes, such as in *Mycobacterium* sp. and in the archaea *M. thermautotrophicus* [26]. Despite the absence of these genes in the symbiont, the host trypanosomatid presents both PIS and inositol membrane transporter genes, as supported by the transcriptome data of *A. deanei* (submitted to publication), indicating that the bacterium obtains its PI directly from the host. Taking these data together, we can suggest that the symbiotic association maintains the optimal lipid content: the prokaryote stimulates the glycerophospholipid metabolism in the protozoan, while the host supplies the symbiont with key phospholipids.

The occurrence of gene transfer has been previously described in *A. deanei*. Analysis of biosynthetic routes revealed that many horizontal gene transfers occurred from diverse bacterial lineages to the nucleus, thus indicating previous symbiotic associations in trypanosomatids. Some of these transferred sequences originated from the present endosymbiont [3]. However, in the present work, gene searches and phylogenetic analyses have never grouped trypanosomatid sequences with any bacterial ortholog, revealing that PIS, MIPS, and the inositol membrane transporter sequences found in *A. deanei* and other trypanosomatids have eukaryotic origin and are not derived from bacterial gene transfers.

## Conclusions

In this work, we showed that *A. deanei* is able to synthesize PI and phosphoinositides from [^3^H]*myo*-inositol incorporation, which was corroborated by genomic and phylogenetic data. The presence of the symbiotic bacterium does not influence the [^3^H]*myo*-inositol incorporation by the host trypanosomatid. The isolated symbiont is not able to produce its own PI, which is evidenced by the lack of sequences coding enzymes and proteins for PI synthesis in its genome. The overall data provide strong evidence for the view that PI is produced by the host and further incorporated, processed, and metabolically employed by the bacterium in its biological processes. We also suggest that the above mentioned PI metabolism route, shared in this mutualistic association, would represent a mechanism used by the host to control the bacterium size and division.

## Additional files

Additional file 1: Figure S1. Full version of the molecular phylogenetic analysis of PIS by the maximum likelihood method. Branches were colored based on taxonomic affiliation, according to the legend on the right. Numbers on nodes represent bootstrap support values. The clade comprising the Trypanosomatidae family was represented by thicker branches. Additional file 2: Figure S2. Molecular phylogenetic analysis of the inositol transporter by the maximum likelihood method. Branches were colored based on taxonomic affiliation, according to the legend on the right. Numbers on nodes represent bootstrap support values. The clade comprising the Trypanosomatidae family was represented by thicker branches. Additional file 3: Figure S3. Molecular phylogenetic analysis of MIPS by the maximum likelihood method. Branches were colored based on taxonomic affiliation, according to the legend on the right. Numbers on nodes represent bootstrap support values. The clade comprising the Trypanosomatidae family were represented by thicker branches.

## Abbreviations

AIS: Archaeal inositol synthase
CDP-DAG: Cytidine diphosphate-diacylglycerol
ER: Endoplasmic reticulum
MIPS: L-*myo*-inositol 1-phosphate synthase
PC: Phosphatidylcholine
PE: Phosphatidylethanolamine
Pi: Inorganic phosphate
PI: Phosphatidylinositol
PI-K: Phosphatidylinositol kinase
PI-PLC: Phosphatidylinositol phospholipase C
PIP: Phosphatidylinositol monophosphate
PIP2: Phosphatidylinositol biphosphate
PIP3: Phosphatidylinositol triphosphate
PIS: Phosphatidylinositol synthase
