# Supplementary figures and images for: Biochemical and phylogenetic analyses of phosphatidylinositol production in Angomonas deanei, an endosymbiont-harboring trypanosomatid
Source: Parasit Vectors. 2015 Apr 24;8:247. doi: 10.1186/s13071-015-0854-x (PMC4424895; doi:10.1186/s13071-015-0854-x)

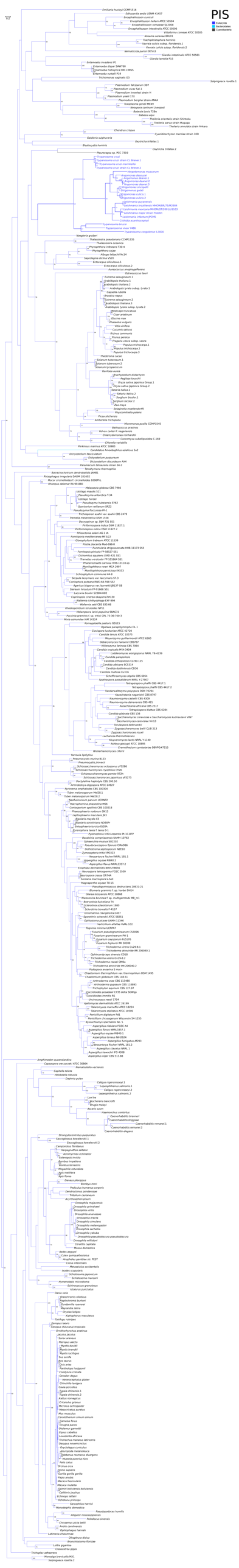

Supplement: Additional file 1: Figure S1. — Full version of the molecular phylogenetic analysis of PIS by the maximum likelihood method. Branches were colored based on taxonomic affiliation, according to the legend on the right. Numbers on nodes represent bootstrap support values. The clade comprising the Trypanosomatidae family was represented by thicker branches. [file 13071_2015_854_MOESM1_ESM.pdf]
