# Supplementary material for: Biochemical and phylogenetic analyses of phosphatidylinositol production in Angomonas deanei, an endosymbiont-harboring trypanosomatid
Source: Parasit Vectors. 2015 Apr 24;8:247. doi: 10.1186/s13071-015-0854-x (PMC4424895; doi:10.1186/s13071-015-0854-x)
Supplement: Additional file 3: Figure S3. — Molecular phylogenetic analysis of MIPS by the maximum likelihood method. Branches were colored based on taxonomic affiliation, according to the legend on the right. Numbers on nodes represent bootstrap support values. The clade comprising the Trypanosomatidae family were represented by thicker branches. [file 13071_2015_854_MOESM3_ESM.pdf]

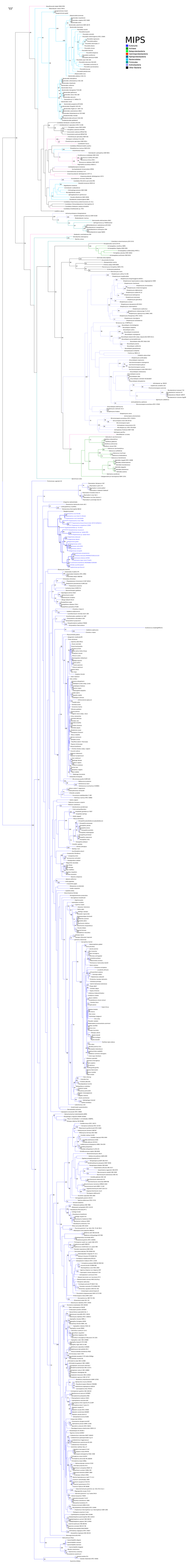
MIPS
Eukaryota
Archaea
Betaproteobacteria
Gammaproteobacteria
Alphaproteobacteria
Bacteroidetes
Firmicutes
Actinobacteria
Other Bacteria
